# Phase separation in cancer at a glance

**DOI:** 10.1186/s12967-023-04082-x

**Published:** 2023-04-01

**Authors:** Qingqing Xie, Jiejuan Cheng, Wuxuan Mei, Dexing Yang, Pengfei Zhang, Changchun Zeng

**Affiliations:** 1 Department of Medical Laboratory, Shenzhen Longhua District Central Hospital, Guangdong Medical University, Shenzhen, 518110 China; 2 School of Pharmacy, Hubei University of Science and Technology, Xianning, 437100 Hubei China; 3 Xianning Medical College, Hubei University of Science and Technology, Xianning, 437100 Hubei China

**Keywords:** Phase separation, Cancer, Mechanism, Cancer biology, Therapy

## Abstract

Eukaryotic cells are segmented into multiple compartments or organelles within the cell that regulate distinct chemical and biological processes. Membrane-less organelles are membrane-less microscopic cellular compartments that contain protein and RNA molecules that perform a wide range of functions. Liquid–liquid phase separation (LLPS) can reveal how membrane-less organelles develop via dynamic biomolecule assembly. LLPS either segregates undesirable molecules from cells or aggregates desired ones in cells. Aberrant LLPS results in the production of abnormal biomolecular condensates (BMCs), which can cause cancer. Here, we explore the intricate mechanisms behind the formation of BMCs and its biophysical properties. Additionally, we discuss recent discoveries related to biological LLPS in tumorigenesis, including aberrant signaling and transduction, stress granule formation, evading growth arrest, and genomic instability. We also discuss the therapeutic implications of LLPS in cancer. Understanding the concept and mechanism of LLPS and its role in tumorigenesis is crucial for antitumor therapeutic strategies.

## Introduction

The execution of biological functions depends on the coordination of complex biochemical events in a dense cellular space. Cancer is known to cause widespread disruption to the regulation of various cellular processes. These processes include transcription, chromatin organization, RNA processing, genomic integrity, and signaling, which are responsible for diseases characterized by unregulated cell proliferation and growth, enhanced cell survival, and metabolic reprogramming [1]. The molecular mechanism by which coordination occurs in biological pathways may shed light on the molecular basis of cancerous pathway disruption. Eukaryotic cells evolved unique inner structures, compartments, or organelles with specific properties and functions to orchestrate biochemical reactions. Typically, lipid bilayer membranes separate the exterior from the interior environment of a membrane-bound organelle, such as the nucleus, endoplasmic reticulum, lysosomes, mitochondria, secretory vesicles, and Golgi apparatus [2].

In eukaryotic cells, biomolecular condensates (BMCs) are membrane-less assemblies that play a crucial role in many cellular functions via the compartmentalization of specific nucleic acids and proteins within subcellular compartments. Membrane-less bodies, including leukopoietins, nucleosomes, parameres, and Cajal bodies, stress granules (SGs), signaling sites, and processing bodies, are involved in signaling, punctum formation, and transduction, ribosome biogenesis, and cell division [3]. The formation of BMCs can be attributed to weak, multivalent interactions between macromolecules such as proteins and nucleic acids [4, 5]. Increasing evidence suggests that BMCs are dynamically and reversibly assembled through liquid–liquid phase separation (LLPS) [6]. Various components of functional membrane-less organelles exist as liquid-like droplets [7]. Cells have evolved multiple mechanisms to maintain LLPS. Intrinsically disordered regions (IDRs), proteins with multiple folded domains, metal ion-controlled RNA-binding domains and nucleic acid chains can drive LLPS through their multivalency [6, 8]. Metal ions are also involved in the formation of BMCs through LLPS [8]. A dysfunctional LLPS leads to tumorigenesis through the disruption of BMCs [9]. Here, we summarize recent insights and findings on the formation, regulation, function, and mechanism of LLPS. In addition, we present how LLPS contributes to the development of cancer. Finally, we describe how innovative cancer therapies may benefit from the regulation of the LLPS.

## Biological characteristics of LLPS

LLPS occurs within cells as macromolecules transition between dense and diluted phases, which is necessary for achieving the lowest free energy state [10]. A characteristic of LLPS is the presence of threshold concentrations of macromolecules, which are affected by several biophysical parameters, including temperature, salt concentration, and ions [6]. Proteins, DNA, and RNA, which are the primary elements and mediators of LLPS, may interact to create a highly multi-component system. Multivalent interactions exhibit high affinity and stereospecificity, which enables the assembly of oligomers and polymers. A higher valency allows for the development of larger oligomers or polymers at lower saturation levels. Nucleic acid chains, IDRs, and multiple folded domains can all result in multivalency in proteins [11]. The LLPS process can produce several states, including liquid droplets, hydrogels, and fibrous aggregates. In some cases, the aggregation state can affect the protein function [3]. Scaffold molecules are composed of multivalent nucleic acids or proteins that are critical for the initiation of LLPS. The surfaces of macromolecules will be subjected to transient, weak, nonspecific interactions with their counterparts in the cytoplasm or nucleoplasm [12]. Many structural characteristics are observed in proteins undergoing LLPS, such as repetitive modular domains, weakly adhesive multivalent motifs, IDRs, nucleic acid recognition domains, and oligomerization or dimerization domains [13]. Many RNA-binding proteins possess IDRs and low complexity domains (LCDs), known as prion-like domains, which allow the formation of BMCs in a highly crowded nuclear environment [14]. BMCs can be facilitated by weak interactions, such as hydrophobic, electrostatic, cation–pi, and pi–pi interactions [15].

**Fig. 1:**
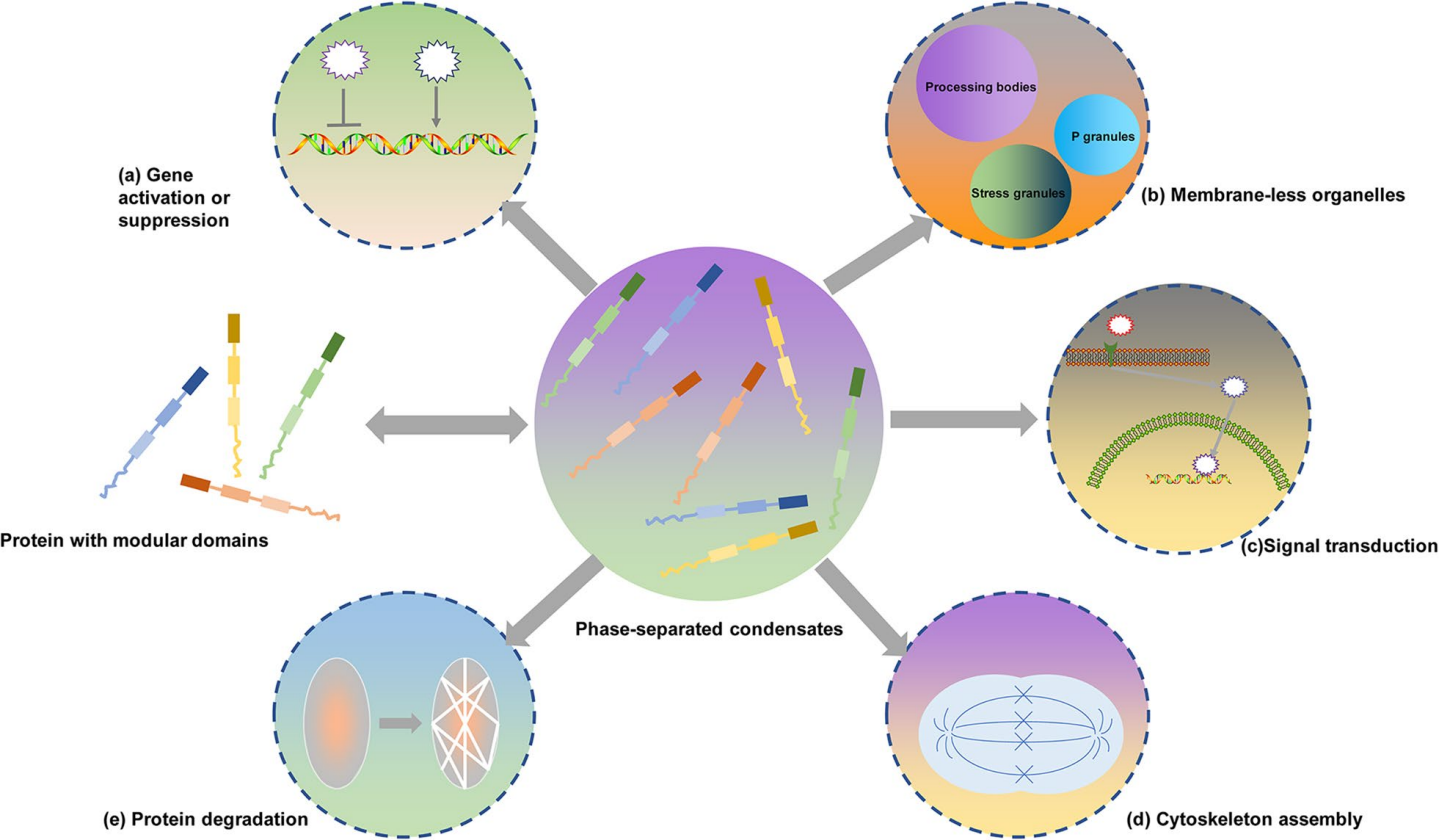
illustrates the biological processes involved in LLPS. **a** The activation or suppression of genes, including transcription, epigenetics, and translation. **b** Processing bodies, Stress granules, and P granules are formed as membrane-less organelles. **c** The process of signal transduction. **d** Assembly of the cytoskeleton. **e** Degradation of proteins

Protein-dependent LLPSs are biophysical processes that rely heavily on scaffolds and clients for their formation and function, providing a mechanism for regulating and compartmentalizing biochemical processes inside cells (Fig. 1) [9]. Only a few LCDs can maintain a three-dimensional structure, and these regions serve as scaffolds in motif interaction. Typically, the disordered region is highly enriched in specific amino acids containing aromatic residues, charged residues, or hydrophilic residues, all of which are associated with pi–pi, cation–Pi, and electrostatic interactions among these amino acids. Conversely, aliphatic residues such as isoleucine, leucine, and valine are less common in LCDs [16]. Scaffold molecules in cells are typically highly concentrated and have multiple valences based on the number of disturbing modules [17, 18]. Additionally, post-translational modifications of scaffolds can control the condensate assembly by modulating weak interactions between multivalent molecules that lead to LLPS. The type and state of the scaffold protein, the recruited client cells, and their location can impact the physiological functions of BMCs [17]. A variety of forces are simultaneously involved in motif interactions and driving LLPS, such as sheets, electrostatic interactions (cation-anion), Van der Waals forces (dipole–dipole), and -effects (cation–, ‑stacking) [13, 17, 19]. Proteins with repetitive sequences of proline-rich motifs and Src homology 3 domains can also serve as endogenous LLPS through concentration-dependent mechanisms. RNA can participate in the cellular development of RNA/protein-rich membrane-less aggregates by facilitating LLPS. A low ratio of RNA to protein can facilitate LLPS droplet formation, whereas a high RNA to protein ratio can inhibit it. A reduction in nuclear RNA levels or a genetic alteration that affects RNA binding within cells can result in LLPS, which may cause aggregate formation [19].

## Liquid–liquid phase separation in cancer

**Fig. 2:**
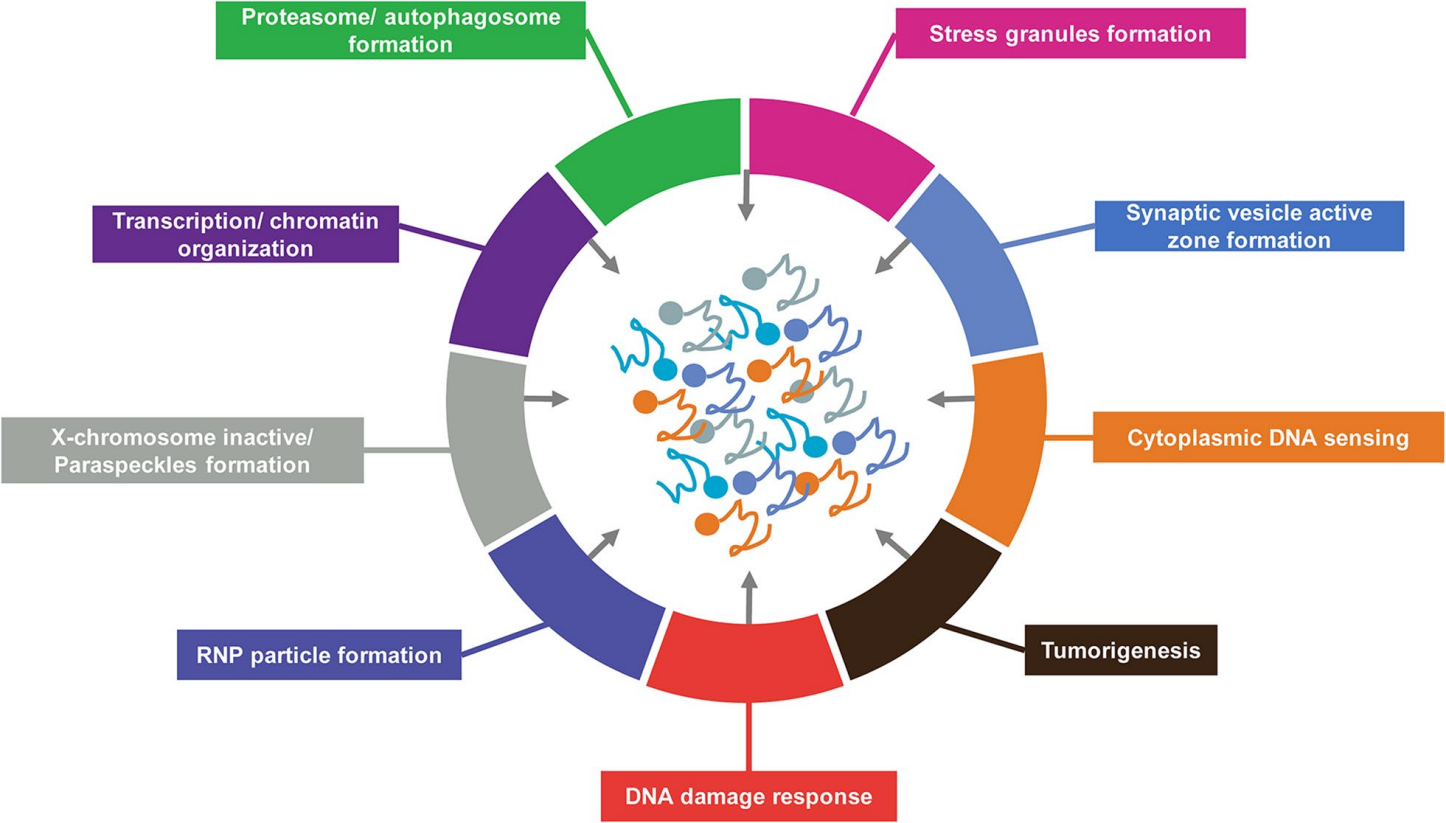
An overview of the biological functions of LLPS-mediated membrane-less biomolecular condensates is presented, including X-chromosome inactivation/paraspeckle formation, transcription/chromatin organization, cytoplasmic DNA sensing, DNA damage response, stress granule formation, proteasome/autophagosome formation, tumorigenesis, synaptic vesicle active zone formation, and ribonucleoprotein synthesis

In recent decades, tremendous progress has been made in understanding malignant tumors. Cancer cells are characterized by several traits, including sustained proliferative signaling, resistance to cell death, induction of angiogenesis, replication of immortality, evasion of growth suppressors, and activation of invasion and metastasis [20]. In addition, new hallmarks of cancer have emerged, including the deregulation of cellular metabolism, genome instability, mutation, senescent cells, polymorphic microbiomes, tumor-promoting inflammation, replication immortality, immunity to destruction, and non-mutational epigenetic reprogramming [21]. Despite this, the mechanism by which cancer develops remains unclear. Numerous studies have established the crucial contribution of LLPS to cancer, which regulates various biological processes such as X-chromosome inactivation/paraspeckle formation, transcription/chromatin organization, cytoplasmic DNA sensing, DNA damage response, SGs formation, proteasome/autophagosome formation, tumorigenesis, synaptic vesicle active zone formation, and ribonucleoprotein (RNP) synthesis (Fig. 2) [22]. LLPS provides new perspectives for understanding cancer development and may facilitate the development of potential therapeutic strategies.

## Misregulated signaling and transduction

Cancer-related mutations can trigger the formation of BMCs. LLPS facilitates signaling condensate formation and modulates immune signaling pathways that include the downstream of B cell receptor, T cell receptor, as well as innate immune receptors such as retinoic acid-inducible gene I protein (RIG-I) and cyclic GMP-AMP synthase (cGAS)-stimulator of interferon genes (STING) [23]. Neurofibromin 2 (NF2) stimulates innate immunity through the regulation of YAP/TAZ-mediated inhibition of tank-binding kinase 1 (TBK1). NF2 mutants suppress cyclic GMP-AMP synthase (cGAS)-stimulator of interferon genes (STING) and RIG-I-like Receptor (RLR)-mitochondrial antiviral signaling protein (MAVS) signaling. LLPS of NF2 mutants impairs antitumor immunity induced by STING [24]. 2′3′-cGAMP (cyclic GMP-AMP) causes the LLPS of endoplasmic reticulum (ER)-associated STING to generate BMCs. STING BMCs restrict the overactivation of innate immunity through the separation of STING-TBK1 and IRF3 [25]. As a result of LLPS, membrane receptors and their signaling molecules can self-assemble during signaling, such as during T-cell receptor (TCR) signaling in immune cells. In response to TCR activation, tyrosine residues in LA were phosphorylated by the zeta chain of TCR-associated protein kinase 70 (ZAP70) [26]. As a result, the formation of condensation is induced by the attraction of multivalent SH-containing proteins, such as growth factor receptor-bound protein 2 (GRB2), phospholipase C (PLC), and GRB2-related adaptor downstream of Shc (GADs). Following this, SOS1 is recruited to participate in the activation of RAS signaling [27]. Furthermore, the SH2 domain-containing leukocyte protein of 76 kDa (SLP76) attaches to GRB2 or GADs and recruits the noncatalytic region of tyrosine kinase (NCK) and neural Wiskott-Aldrich syndrome protein (N‑WASP) actin effectors and the actin-related protein 2/3 (ARP2/3) complex that is necessary to assemble actin filaments. LLPS prevents spontaneous SOS1 membrane localization from membrane-dependent RAS activation, thus initiating RAS signaling. Nevertheless, oncoproteins of the chimeric receptor tyrosine kinases (RTKs) impair RAS signaling mediated by LLPS [26, 28].

Mutant oncoproteins exhibit LLPS due to their multivalent interactions. In the case of echinoderm microtubule-associated protein-like 4 (EML4)-anaplastic lymphoma kinase (ALK) or coiled-coil domain-containing protein 6 (CCDC6)-rearranged during transfection (RET), multimerization domains of EML4 or CCDC6 were assembled, whereas targeting sequences of ALK or RET were lost, resulting in the production of granules without membranes [13, 29]. As a result, these granules can create concentrations of the RAS-activating complex GRB2/son of sevenless 1 (SOS1), thereby facilitating RAS signaling independent of the membrane lipids. LLPS is enabled by mutant SH2 domain-containing protein tyrosine phosphatase 2 (SHP2) with an open conformation in its PTP domain. Oncogenic SHP2 mutants activate RAS-MAP kinase signaling through LLPS by recruiting wild-type SHP2 into BMCs [30].

The 3′,5′-cyclic adenosine monophosphate (cAMP)-dependent protein kinase A (PKA) signaling transduction further reveals that LLPS facilitates the rapid concentration of critical signaling pathway components in the cytoplasm in cancer cells. PKA, a tetrameric holoenzyme, is composed of a dimer of regulatory subunits and a pair of catalytic subunits. The non-redundant regulatory subunit RI, a cAMP receptor, is essential for the function of PKA [31, 32]. In normal cells, the RI can contribute to BMCs that can sequester high concentrations of cAMP and maintain high levels of PKA activity. It can also function as a dynamic buffer for cAMP. In atypical liver fibrolamellar carcinoma, the DnaJ Hsp40 member B1 (DnaJB1)-PKAcat fusion proteins replace the native N terminus of PKA-C with the J domain of DnaJB1, which abolishes the RI LLPS, enhances the levels of cAMP in positioned phosphodiesterase sinks, and activates cAMP signaling [31].

Abnormal LLPS are also associated with the dysfunction of signal transduction pathways. Glycogen accumulation occurs in precancerous liver lesions due to the downregulation of the glucose 6-phosphatase (G6PC), which modulates hepatic glycogenolysis. The LLPS then forms condensed glycogen compartments from the accumulated glycogen. Laforin-macrophage stimulating 1/2 (MST1/2) complexes aggregate in glycogen-liquid droplets to relieve MST1/2 inhibition on the yes-associated protein (YAP), enabling tumorigenic cell transformation. The disheveled binding antagonist of -catenin 1 (DACT1), a target gene of transforming growth factor (TGF-), can assemble BMCs within the cytoplasm that sequester casein kinase 2 (CK2) and prevent WNT signaling. Thus, DACT1 BMCs play a vital role in the metastatic process of breast and prostate cancer cells [33, 34]. IDRs within the signalosome scaffold protein Dishevelled 2 (Dvl2) mediate LLPS. The receptor Fzd5, which is a component of the signalosome, is responsible for stimulating LLPS. Dvl2 LLPS can recruit Axin to stabilize β-catenin, which is crucial for the assembly of the Wnt signalosome and the disassembly of the destruction complex [35, 36].

## Stress granules

Under various stress conditions, eukaryotic cells form SGs that halt translation and release mRNA molecules from polysomes. The SGs are cytoplasmic compartments of the RNA granule family that respond to various stress signals and form cytoplasmic BMCs in eukaryotic cells, thereby facilitating cell survival. The assembly of SGs is related to numerous RNA-binding proteins implicated in RNA processing and nuclear transport. Primary SGs are coordinated by the complexes of RNA-binding proteins and mRNA via LLPS [37]. The SG protein G3BP1 enters an auto-inhibited condition in the absence of stress by interacting with its IDRs and arginine-rich regions. Stress conditions cause unfolded mRNAs to liberate G3BP1 from its auto-inhibited conformation, leading to G3BP1 clustering via the interactions between protein and RNA. Subsequently, G3BP1/RNA-phase separated BMCs occurred, and G3BP1 hindered RNA entanglement, recruiting client proteins to facilitate the assembly of SGs [38]. An interaction network between proteins and RNA is responsible for SGs via LLPS. G3BP1 activates RNA-dependent LLPS in response to an increase in the concentration of free RNA. Phosphorylation of IDRs in G3BP1 regulates the formation of LLPS. YB1, a member of the cold shock domain (CSD) family, modulates G3BP1 translation to stimulate SG assembly and cancer metastasis [39]. Furthermore, several types of cancer are associated with DDX3X, a component of SGs, which is an ATP-dependent RNA helicase with the conserved motif Asp–Glu–Ala–Asp (DEAD) [40]. Mutant KRAS tumor cells exhibited a significant increase in SG formation [41, 42]. Extrinsic proteins that interact with G3BP1 can regulate the SGs network. The assembly of RNP SGs is also mediated by heterotypic multivalent interactions [41]. UBQLN2, a shuttle protein for the proteasome, possesses IDRs and is located within the SGs. The oligomerization of UBQLN2 is essential for the occurrence of LLPS. UBQLN2 interacts with ubiquitinated client proteins to facilitate the shuttling of client proteins out of SGs and reverses the UBQLN2-induced LLPS [43].

## Evading growth arrest

Evading growth arrest caused by endogenous tumor suppressors can boost cell growth. There is considerable evidence that the nuclear speckle-type pox virus and zinc finger (POZ) protein (SPOP), which serves as a substrate-recognition component of the cullin-RING E3 ligase (CRL3), appears to play a crucial role in carcinogenesis and cancer development [44]. SPOP is a tumor suppressor protein with an N-terminal MATH domain, an internal BTB domain, and a nuclear localization sequence (NLS) at the C-terminus. Several oncoproteins, such as death domain-associated protein (DAXX), myelocytomatosis oncogene (MYC), androgen receptor (AR), GLI family zinc finger 3 (GLI3), and steroid receptor coactivator 3 (SRC3), can be recruited by SPOP to CRL3 for ubiquitination and degradation by proteasomes [45, 46]. The NLS of SPOP facilitates its localization to nuclear speckles, which are RNA-protein granules implicated in the regulation of gene expression, promyelocytic leukemia (PML) bodies, DNA damage loci, metabolism, and splicing. SPOP can oligomerize to localize nuclear speckles, which increases the efficiency of ubiquitination. SPOP interaction with death domain-associated protein (DAXX) can induce droplet formation through LLPS. SPOP alterations impair LLPS and DAXX ubiquitination, which may lead to oncoprotein accumulation [19, 46, 47]. In membrane-less organelles, mutations in the SPOP lead to ubiquitin-dependent protein homeostasis by disrupting LLPS and colocalization [48].

Studies in vivo indicate that p53 participates in cytosolic droplets, implying that LLPS may be critical for the biological activity of p53 [49]. It has been established that the transcriptional regulator p53 regulates processes, including the cell cycle, apoptosis, and senescence, in response to a wide range of stress signals, such as hypoxia, DNA damage, and oxidative stress [50]. The p53 consists of two transactivation domains (TADs), a sequence-specific DNA-binding domain (DBD), a C-terminal regulatory domain (CTD) associated with nuclear localization, and an oligomerization domain (OD) essential for transcriptional activity. The modulation of droplet formation by truncated TP53 mutant points to the significance of multivalent electrostatic interactions between the N-terminal and C-terminal domains of p53 [49]. P53 BMCs depend on the disordered unstructured basic regions (UBRs) that are regulated by electrostatic and hydrophobic interactions. The tetramerization domain (TD) mutations interfere with LLPS of p53 by blocking the production of tetramers [51]. Oncogenic mutations in TD are responsible for preventing the production of p53 BMCs, which results in decreased target gene activation and accelerates cancer development. The disordered TAD of p53 can regulate LLPS and amyloid aggregation. The DBD of p53 underwent LLPS in the presence of polyethylene glycol. DBD mutants of p53, such as M237I and R249S, also experienced LLPS [52].

## Maintenance of genome stability

DNA damage events triggered by exogenous and endogenous factors may affect genomic instability and cause cancer. DNA damage response (DDR) and DNA repair processes are frequently disrupted by genetic alterations. DNA repair lesions generate transient and reversible BMCs that induce the repair of proteins and the generation of repair signals. A nucleic acid-like protein modification known as poly (ADP-ribose) (PAR) can be found in BMCs, such as DNA repair foci and SGs. LCDs in PAR-binding or PAR-conjugated proteins promote LLPS and the formation of BMCs. PAR promotes LLPS of proteins with LCDs such as TDP-43, FUS, and hnRNPA1 [53].

The poly (ADP-ribose) polymerase 1 (PARP1) is a critical component of the cancer biology process through its involvement in replication, transcription, chromatin remodeling, genome maintenance, and DNA repair [54]. As a poly (ADP-ribosylation) (PARylation) “writer”, PARP1 synthesizes negatively charged PAR chain polymers. As a result of DNA damage, PARylation is believed to control the physicochemical characteristics, assembly, and catalysis of target proteins [55]. The cellular response to DNA damage is characterized by an increase in PAR levels induced by PARP enzyme hyperactivation at sites of DNA damage, leading to the rapid accumulation of proteins with LCDs. LCD-containing proteins experience LLPS and liquid demixing, which allows cells to filter molecular interactions involving damaged chromatin. Upon dissolution of PAR-seeded liquid compartments, interactions unfold on lesion-flanking chromatin, allowing 53BP1 to accumulate. As a modulator of DNA double-strand break (DSB) repair, 53BP1 stimulates the development of chromatin domains surrounding the damaged DNA [56]. A disruption of LLPS of 53BP1 reduces the 53BP1-dependent activation of p53 and weakens the expression of p53 target genes. During LLPS of 53BP1, localized recognition of DNA damage and repair factor assembly is coordinated with p53-dependent gene activation and cell fate determination [57]. Additionally, 53BP1 promotes the integrity of heterochromatin and genome stability through LLPS in addition to its role in DSB repair [58]. By inactivating P-TEFb, a heterodimer of CDK9 and cyclin T1 (CycT1), PARP1 suppresses Pol II elongation. In response to damage, stimulated PARP1 attaches to transcriptionally active P-TEFb and modifies CycT1 at multiple sites of the protein. P-TEFb-PARP1 signaling contributes to the maintenance of genomic stability and the regulation of transcription quality following DNA damage [59].

## Tumor viruses

Evidence suggests that tumor-associated viruses, such as Epstein–Barr virus (EBV) and Kaposi sarcoma herpesvirus (KSHV), regulate the progression of tumors through LLPS [60–62]. KSHV genomes interact with Latency-Associated Nuclear Antigen (LANA) to generate stable nuclear bodies (NBs). The LANA-associated nuclear bodies (LANA-NBs) are dependent on LLPS to develop dynamic structures that vary during the viral life cycle [60]. The LLPS of EBV proteins play a critical role in controlling the expression of host genes. EBV proteins EBNA2 and EBNALP regulate virus and cellular gene transcription as transcription factors, which can form liquid-like BMCs at Runx3 and MYC super-enhancer sites [61]. LLPS of EBNA2 mediates alternative RNA splicing patterns in cancer. EBNA2 modulates the aberrant splicing of MPPE1 through the recruitment of SRSF7 and SRSF1 to its motif [62]. In cells, the human papillomavirus (HPV) E2-p53 interaction involved in viral replication occurs via a direct interaction of proteins that promote heterotypic LLPS [63]. This interaction occurs specifically between the E2 DNA binding domain and the N-terminal transactivation domain of p53.

## Cancer metabolism

LLPS has been identified as a mechanism that regulates the activity of enzymes within cells. Under hypoxia and high energy demands, glycolysis is elevated to stimulate cell proliferation. In hepatocarcinoma cells, hypoxia causes glycolytic enzymes such as the phosphofructokinase subunit Pfk2p to concentrate within membrane-less granules known as the glycolytic (G) body [64]. Snf1p, an AMP-activated protein kinase, is required for the formation of G bodies. G-body formation through LLPS is correlated with enhanced cell survival and proliferation in hypoxic stress conditions. Under hypoxic conditions, RNA is co-localized with glycolytic enzymes to the G bodies [65]. RNA scaffolding and recruitment are required for G body formation. Glycolysis is accelerated by the formation of G bodies through multivalent protein-RNA and protein–protein interactions. Under hypoxia, RNA facilitates the LLPS of glycolysis enzymes into G bodies. Condensed glycogen undergoes LLPS in vivo and in vitro, which results in the Laforin-Mst1/2 complex assembly in the glycogen droplet and triggers Yap. Glycogenolysis enzyme-liver glycogen phosphorylase (PYGL) or glucose-6-phosphatase (G6PC) deficiency results in glycogen storage diseases associated with Yap activation, and tumorigenesis [66].

## LLPS in cancer therapy

With the advancement in knowledge of LLPS biology in cancer, the potential for developing effective cancer therapeutics has been raised, despite the challenges and obstacles that remain. There is potential therapeutic benefit from disrupting condensate formation through IDRs or physicochemical properties. Additionally, it has been proposed that selective modulation of LLPS, such as PTMs, may be an alternative therapeutic approach [18].

**Fig. 3:**
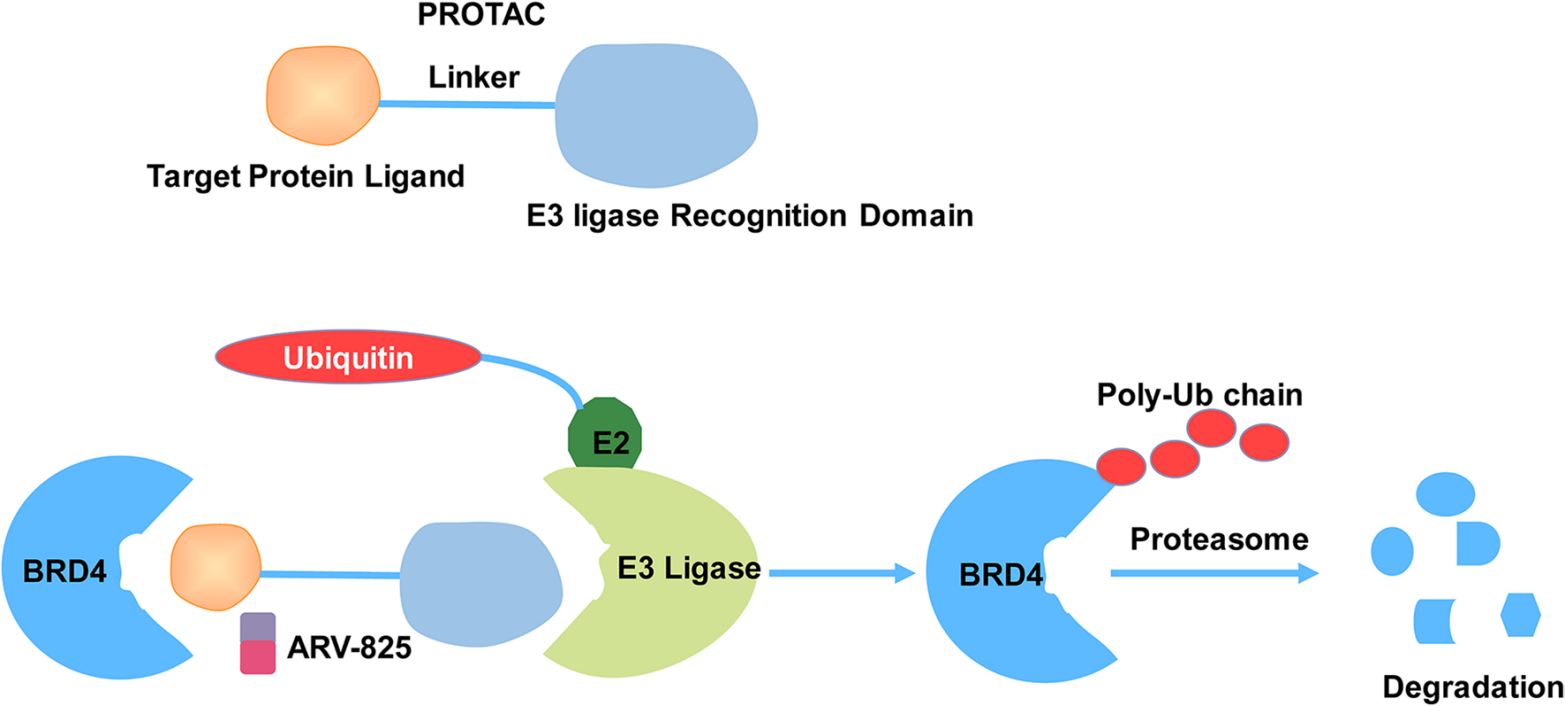
An innovative PROTAC, ARV-825, which links BRD4 to E3 ubiquitin ligase, effectively degrades BRD4 protein. ARV-825-induced BRD4 aggregation disrupts LLPS and suppresses BRD-4-dependent transcription. *PROTAC* proteolysis targeting chimera

**Fig. 4:**
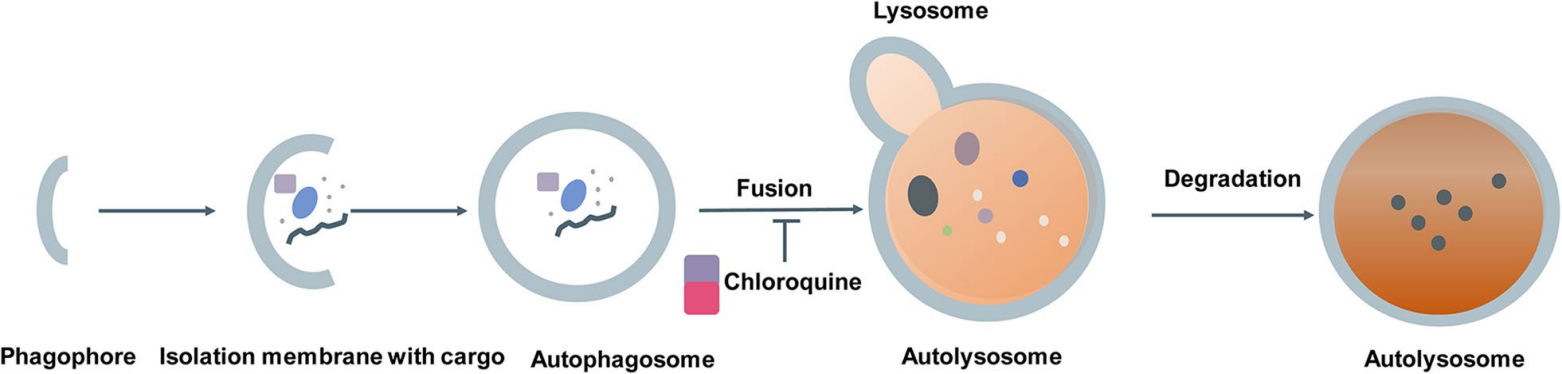
Chloroquine inhibits autophagy by impairing the autophagosome-lysosome fusion process, thereby increasing target protein levels. LLPS regulates the assembly of autophagy substrates

BMCs may form due to the thermodynamic instability of IDRs [6]. However, few IDR-binding compounds have been identified, and little attention has been paid to their ability to destabilize BMCs. Small molecule drugs are concentrated in BMCs, causing alterations in their on-target efficacy and pharmacodynamics [67]. There is increasing evidence that small molecules can bind to the IDRs of transcription factors such as TAF2, MYC, c-FOS, p53, and EWS and inhibit malignant cell transformation [13]. p53 structural mutants cause global destabilization, misfolding, and aggregation of the p53 protein. p53 aggregation can occur through a condensate-like state, and p53 can be found in protein BMCs. Small molecule compounds that interact with the p53 protein can be identified and used to dissolve the BMCs formed by the p53 structural mutant [52, 68]. Unlike p53 aggregation inhibitors, these identified small molecule compounds are efficacious against p53 BMCs and do not cause the reactivation of mutant p53 [68]. Several drugs, such as cisplatin, mitoxantrone, THZ1 (a CDK7 inhibitor), and tamoxifen were identified to selectively partition into BMCs formed by MED1. Mitoxantrone was also accumulated in nucleolar protein BMCs produced by NPM1 and FIB1 [67]. The BRD4 inhibitor was enriched in ED1, BRD4, and NPM1 BMCs [6]. The BET inhibitor that specifically targets BRD4 can operate by decreasing its LLPS [69]. Through the recruitment of BRD4 to the E3 ubiquitin ligase cereblon, ARV-825 (a BRD4 degrader) can induce efficient and sustained degradation of BRD4 in Burkitt’s lymphoma cell lines. ARV-825, a hetero-bifunctional proteolysis-targeting chimera (PROTAC), exerts a more effective inhibition of c-MYC levels and downstream signaling than small-molecule BRD4 inhibitors in Burkitt’s lymphoma cell lines, resulting in a significant decrease in cell proliferation and an increase in apoptosis (Fig. 3) [70, 71]. Chloroquine inhibits autophagy by impairing the autophagosome-lysosome fusion process, thereby increasing target protein levels (Fig. 4). Moreover, chloroquine prevents SQSTM1-positive autophagosomes from fusing with lysosomes, resulting in the buildup of STX17 [72].

Posttranslational modifications (PTMs) including arginine methylation, phosphorylation, arginine citrullination, ubiquitination, acetylation, and poly(ADP-ribosyl)ation mediate the condensation process and impact the properties of membrane-less compartments [73]. PTMs of RBPs as significant regulators of LLPS and RNP granule dynamics can decrease or strengthen the multivalent interactions between macromolecules as well as either exclude or recruit specific macromolecules from or into BMCs [74]. In the LLPS and RNP granule dynamics, arginine-methylation and phosphorylation function as important PTMs [75]. Assembling and disassembling SGs are regulated by deubiquitylases and small ubiquitin-like proteins, such as NEDD8 and SUMO [76]. Ubiquitin and ubiquitin-like proteins bind to target proteins covalently, resulting in a modified binding surface and protein interactions. Ubiquitin interacts with the UBA domain of ubiquitin 2 (UBQLN2), causing UBQLN2 BMCs to disperse [74].

Autophagy-initiated Atg1 complexes generate droplets via LLPS. Point mutations or phosphorylations that impede LLPS reduce the formation of pre-autophagosomal structure (PAS), a liquid-like condensate of Atg proteins [77]. Active TORC1 phosphorylates autophagy proteins, including Atg13, under high nutrient supply, disrupting the Atg13–Atg17 interaction and preventing the formation of PAS. In the absence of nutrients, TORC1 dephosphorylates autophagy proteins, including Atg13, which aggregates Atg1 molecules and autophosphorylates them. By activating Atg1, Atg13 can be re-phosphorylated, and PP2C phosphatases can reverse this process, thereby contributing to PAS condensate stability [78]. PAR polymerase (PARP) can regulate PARylation, and poly(ADP-ribose) glycohydrolase (PARG) hydrolyzes PAR to separate damaged DNA compartments. Following PARP1 activation, FUS is directed to sites of DNA damage where it forms the PARG reversible compartment. PARP1 inhibition hinders the formation of DNA damage repair foci and impedes the DNA damage repair process [79].

Various antitumor agents can form BMCs through physicochemical interactions, resulting in therapeutic benefits or drug resistance. The concentration and pharmacodynamics of drugs are affected by condensate characteristics. Antitumor agent cisplatin selectively concentrates in BMCs, promoting disease therapeutic progress [67, 80]. Tamoxifen, a common drug used to treat estrogen receptor (ER)-positive breast cancer, causes the expulsion of ERα from the MED1 BMCs [81, 82]. The ERα concentrates into the BMCs of MED1 in breast cancer cells in a tamoxifen-dependent, estrogen-dependent manner [67].

Some drugs that target transcription factors, hormone receptors, and nucleotide-binding proteins have the potential to prevent disease by inhibiting the formation of BMGs [18, 67]. Due to the heterogeneous and dynamic nature of IDR conformation, administering drugs against them can be challenging. Recently, new developments have been made in drugs that target IDRs. EPI-001 inhibits the androgen receptor by binding to its transduction unit 5 region (Tau-5) thereby delaying the onset of castration-resistant prostate cancer [83]. By controlling LLPS of the prion N-terminal domain in cancer, melatonin can suppress conformational modifications that can lead to aggregation, thus improving multidrug resistance [84].

## Conclusion

Recent studies have demonstrated that BMCs play an important role in cellular processes. BMCs, formed by LLPS and membrane-free organelles, underlie biological processes and reactions. LLPS between macromolecules occurs as a result of multivalent interactions between macromolecules through modular domains, IDRs, and nucleic acid chains. LLPS is crucial in signal transduction, chromosomal disorganization, and transcriptional dysregulation. LLPS can be disrupted by gene mutations or epigenetic variants. LLPS can be governed by determining macromolecule concentration, targeting the components, or interfering with PTMs. Cancer-related molecules in BMCs are identified, and their modulation by LLPS may affect their function and progression. The mechanism responsible for the dynamic properties of LLPS in tumorigenesis and progression is not well understood. Studying LLPS in cancer progression will aid us in understanding the complex pathological processes of cancer and provide new treatment options.

## Data Availability

Data sharing is not applicable to this article as no datasets were generated or analyzed during the current study.

## Abbreviations

LLPS: Liquid–liquid phase separation
TCR: T-cell receptor
ZAP70: Zeta chain of TCR-associated protein kinase 70
GRB2: Growth factor receptor-bound protein 2
PLC: Phospholipase C
GADs: GRB2-related adaptor downstream of Shc
SLP76: SH2 domain-containing leukocyte protein of 76 kDa
NCK: Noncatalytic region of tyrosine kinase
N‑WASP: Neural Wiskott-Aldrich syndrome protein
ARP2/3: Actin-related protein 2/3
RTKs: Receptor tyrosine kinases
EML4: Echinoderm microtubule-associated protein-like 4
ALK: Anaplastic lymphoma kinase
CCDC6: Coiled-coil domain-containing protein 6
RET: Rearranged during transfection
SOS1: Son of sevenless 1
SHP2: SH2 domain-containing protein tyrosine phosphatase 2
cAMP: 3′,5′-Cyclic adenosine monophosphate
PKA: Protein kinase A
DnaJB1: DnaJ Hsp40 member B1
G6PC: Glucose 6-phosphatase
MST1/2: Laforin-macrophage stimulating 1/2
DACT1: Disheveled binding antagonist of -catenin 1
TGF-: Transforming growth factor
CK2: Sequester casein kinase 2
SGs: Stress granules
DEAD: Conserved motif Asp–Glu–Ala–Asp
SPOP: Speckle-type pox virus and zinc finger (POZ) protein
CRL3: Cullin-RING E3 ligase
NLS: Nuclear localization sequence
DAXX: Death domain-associated protein
MYC: Myelocytomatosis oncogene
AR: Androgen receptor
GLI3: GLI family zinc finger 3
SRC3: Steroid receptor coactivator 3
PML: Promyelocytic leukemia
DBD: DNA-binding domain
CTD: C-terminal regulatory domain
OD: Oligomerization domain
UBR: Unstructured basic region
TD: Tetramerization domain
DDR: DNA damage response
PAR: Poly (ADP-ribose)
PARP1: Poly (ADP-ribose) polymerase 1
LCD: Low complexity domain
DSB: DNA double-strand break
CycT1: Cyclin T1
PROTAC: Proteolysis-targeting chimera
PTMs: Posttranslational modifications
RNP: Ribonucleoprotein
UBQLN2: UBA domain of ubiquitin 2
PAS: Pre-autophagosomal structure
ER: Estrogen receptor
Tau-5: Transduction unit 5 region
